# Supplementary material for: Comprehensive Annotation and Functional Exploration of MicroRNAs in Lettuce
Source: Front Plant Sci. 2021 Dec 24;12:781836. doi: 10.3389/fpls.2021.781836 (PMC8739914; doi:10.3389/fpls.2021.781836)
Supplement: Supplementary file 3 [file Data_Sheet_9.ZIP › GSM2230753.plot/Lsa-miRN1655_Lsat_1_v5_gn_6_2461.1_265_TPlot.pdf]

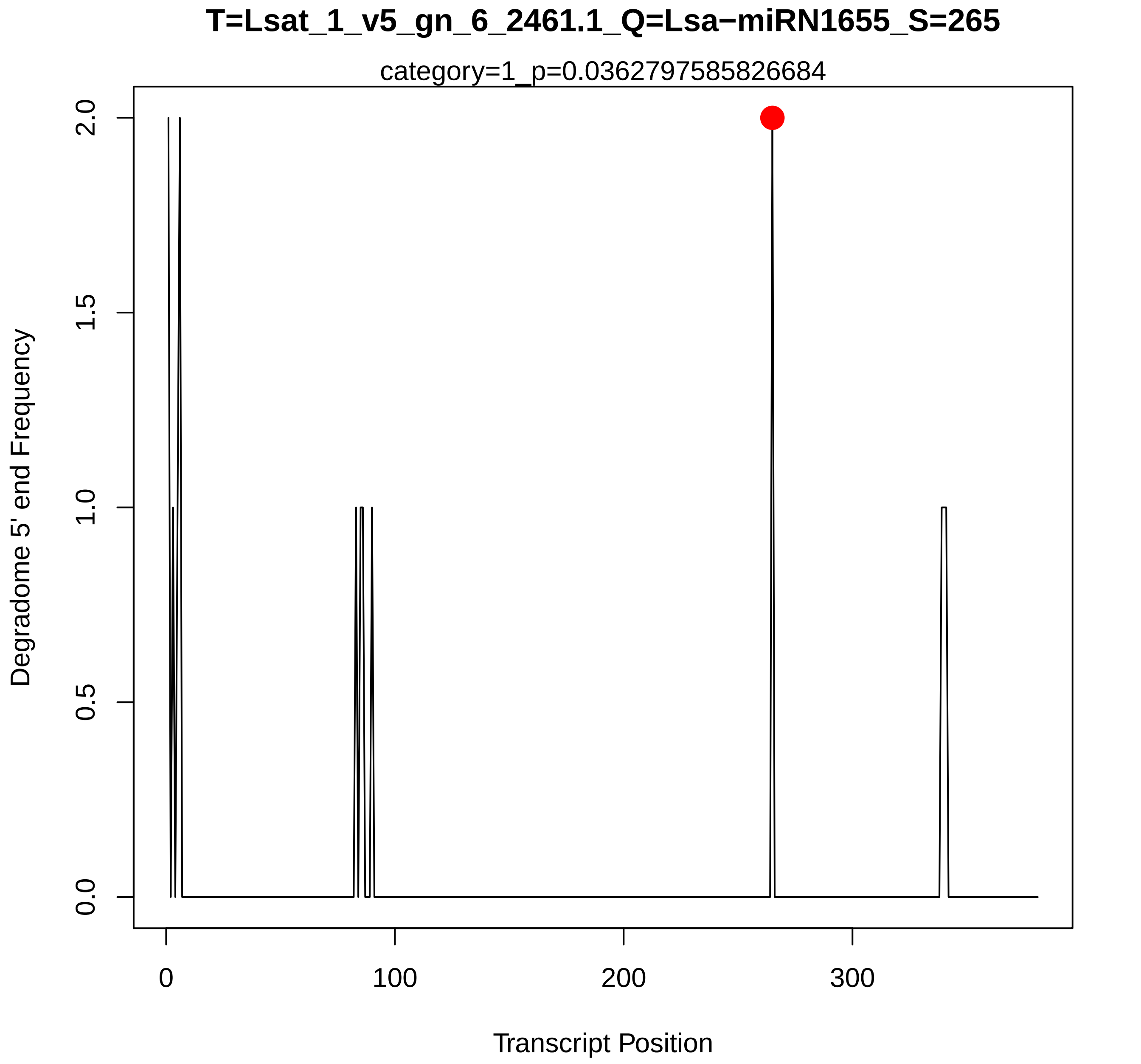
T=Lsat_1_v5_gn_6_2461.1_Q=Lsa−miRN1655_S=265
category=1_p=0.0362797585826684
Degradome 5' end Frequency
Transcript Position
0.0
0.5
1.0
1.5
2.0
0
100
200
300
